# Cannabidiol (CBD) Protects Adipose-Derived Mesenchymal Stem Cells (ASCs) against Endoplasmic Reticulum Stress Development and Its Complications

**DOI:** 10.3390/ijerph191710864

**Published:** 2022-08-31

**Authors:** Anna Kowalczuk, Krzysztof Marycz, Katarzyna Kornicka-Garbowska, Justyna Kornicka, Magdalena Bujalska-Zadrożny, Sylwia Groborz

**Affiliations:** 1 National Medicines Institute, 00-725 Warsaw, Poland; 2 International Institute of Translational Medicine, 55-114 Wisznia Mała, Poland; 3 Department of Experimental Biology, Faculty of Biology and Animal Science, Wrocław University of Environmental and Life Sciences, 50-375 Wrocław, Poland; 4 Faculty of Electronics, Photonics and Microsystems, Wrocław University of Science and Technology, 50-372 Wrocław, Poland; 5 Department of Pharmacodynamics, Centre for Preclinical, Research and Technology (CePT), Medical University of Warsaw, 02-097 Warsaw, Poland

**Keywords:** adipogenesis, adipose stem cells, CBD, ER stress, apoptosis, oxidative stress

## Abstract

Background: Recent studies suggested that individuals with metabolic disorders have altered function of adipocytes and adipose stem cell subpopulations, which impairs tissue homeostasis, promoting insulin resistance and diabetes development. The non-psychoactive phytocannabinoid CBD was found to modulate adipose tissue metabolism, however, its exact role in controlling ASCs’ fate is still poorly understood. Objectives: This investigation aimed to elucidate whether pretreatment of ASCs with CBD can protect against ER stress development and maintain the cytophysiological properties of cells. Methods: Human ASCs were cultured under control and adipogenic conditions. Prior to the experiments, cells in the experimental group were pretreated with CBD following the addition of an ER stress inducer—tunicamycin. After the experiments, the cells were subsequently tested for expression of the apoptotic, ER stress, and anti-inflammatory-related genes using RT-qPCR. Oxidative stress was analysed with flow cytometric assays. Results: Cells pretreated with CBD displayed decreased apoptosis and enhanced proliferation rate. Additionally, the expression of pro-inflammatory cytokines and miRNAs was significantly reduced. The obtained results also demonstrated an obvious reduction in intracellular accumulated ROS and NO, as well as mitigated ER stress through the down-regulation of *IRE-1*, *PERK*, *CHOP*, and *ATF6* transcripts upon CBD treatment. Conclusion: The presented data provide the evidence that CBD protects ASCs against ER stress development and its complications and, thus, offers new insights for the management of obesity through the regulation of adipose tissue dynamics.

## 1. Introduction

Nowadays, adipose tissue (AT) is recognised as an active endocrine organ that, via secretion of a wide range of adipokines, regulates whole-body metabolic homeostasis, including energy balance [1]. These signalling molecules target and modulate functions of many organs including liver, pancreas, brain, skeletal muscle, pancreas, and adipose tissue itself. It is known that AT dysfunction is associated with multiple pathophysiologies including obesity, insulin resistance, type II diabetes, and metabolic syndrome. Substantial research efforts have been undertaken to unravel which of the molecular and cellular cues are associated with increased adiposity and impaired glucose homeostasis, and recently a few adipose-tissue centric mechanisms have been introduced as potential key players. For instance, excessive calorie intake leads to disruption of AT dynamics by triggering hypertrophy (increase in adipocyte size) and hyperplasia (increase in adipocyte number) [2]. Subsequently, AT becomes infiltrated with immune cells which initiates its chronic, low-grade inflammation. As a consequence, impaired glucose and lipid homeostasis as well as energy storage capacity drives the numerous metabolic pathophysiologies.

The unfriendly microenvironment of AT negatively affects the distinct cell populations that reside within it, including mesenchymal stem cells (MSCs) [3,4]. MSC from human AT (adipose stem cells, ASCs) were first described in 2001, and since then have attracted much attention for their unique properties and therapeutic potential. Due to the relatively non-invasive, safe procedure of isolation, ASCs are considered as a go-to agent for tissue engineering, due to their great yield and high proliferation rate, possessing a vast application and research value. Due to extensive, worldwide research on ASCs, in 2006, the International Society for Cellular Therapy has established the minimum criteria for defining these and other types of MSCs, which include (i) adherence to plastic in culture conditions, (ii) expression of CD105, CD73, and CD90 surface antigens, (iii) lack of CD45, CD34, CD14, or CD11b, CD79a, or CD19 and HLA class II expression, and (iv) multipotency, e.g., the ability to differentiate into mesenchymal lineages such as osteoblasts, chondroblasts, and adipocytes [5]. In a healthy state, ASCs participate in AT plasticity via differentiation and secretion of growth factors/cytokines, which modulate their metabolism. However, in a pathophysiological state, these cells suffers from metabolic impairment, which worsens or drive disease progression [6,7]. Disbalance in ASCs’ subtypes, senescence, ageing, and accumulation of oxidative stress factors fail to exert their physiological role in vivo, so their long-term culture in vitro usually is not successful enough to utilise them in autologous therapy. Thus, despite their great therapeutic potential, certain issues need to be addressed before they can be applied in a clinical setting. New strategies, which target and enhance the regenerative properties of ASCs in vitro and in vivo, should be developed in order to assure a successful therapy of metabolic disorders. Multiple rejuvenation methods have been introduced during the past years and have been shown to be safe and effective. Our own research showed that a combination of selected bioactive factors (5-azacytydine and resveratrol) can reverse the ageing of metabolically impaired ASCs in vitro and that these cells exert enhanced therapeutic outcome in vivo [8,9,10].

The endocannabinoid system (ECS) is comprised of molecules that participate in the activity control of the two major cannabinoid lipid-mediators—anandamide (AEA) and 2-arachidonoylglycerol (2-AG). Alternations in their activity have been connected with multiple disorders. ECS is also implicated in the regulation of adipose tissue metabolism, via activation of the cannabinoid receptors called CB1 and CB2 [11]. However, during metabolic disorders, overstimulation of the ECS leads to multiple complications including adipocyte hypertrophy, inflammation, reduced energy expenditure, and β-cell loss. It was shown that CB1 and the endocannabinoid system in human adipose tissue is up-regulated in states of insulin resistance, including diabetes and glucocorticoid exposure, and is related to the diminished ability of developing adipose tissue to take up glucose [12]. As ASCs express CB1 and CB2, the ECS system’s modulation can regulate their migration, survival, differentiation, and, in consequence, influence adipose tissue plasticity.

Phytocannabinoids, which include a class of compounds identified and isolated from *Cannabis sativa*, have been proposed to interact with a variety of molecular targets including CB1 and CB2. In addition, exogenous cannabinoids and their synthetic counterparts are currently used as a therapeutic intervention against a number of diseases such as multiple sclerosis and osteoporosis [13,14]. Among the cannabinoid compounds, the non-psychoactive cannabidiol (CBD) has gained special interest. Unlike classical cannabinoids, CBD lack adverse psychoactive effects, so it can be applied in a clinic. Recent studies indicated that CBD inhibits bone resorption [15], decreases expression of the activator of nuclear factor-κB ligand RANKL/RANK and pro-inflammatory cytokines including interleukin IL-1β and tumour necrosis factor TNF-α) during experimental periodontitis [16], and acts as an anti-arthritic therapeutic [17]. What is more, CBD was shown to promote browning in 3T3-L1 adipocytes, which suggest its potential application as a strategy against obesity development. Additionally, CBD derivative has been recently shown to reduce inflammation as well as protect liver, pancreas, and adipose tissue in mouse models of prediabetes and nonalcoholic fatty liver disease [18].

Here, we introduce an alternative strategy to overcome issues regarding the molecular impairment of ASCs, which consists of their preconditioning in vitro with a cannabinoid compound, cannabidiol (CBD), in order to protect the cells against ER stress development and its molecular consequences.

## 2. Materials and Methods

### 2.1. Experimental Model Setting

Human-adipose-derived stem cells (HuASCs) were isolated from healthy individuals and characterised as described previously [6]. Prior to the experiments, cells were seeded onto 24-well plates at a density of 20 × 10^4^/per well. Cell cultures were pretreatment with CBD (1089161, Sigma-Aldrich/Merck, Poznan, Poland) at a concentration of 5 µM. After 24 h incubation, the medium was changed to medium containing 5 mmol/mL tunicamycin (T7765, Sigma-Aldrich/Merck, Poznan, Poland). Prepared cells were used for further experiments.

For adipose differentation, HuASCs were cultured in commercially available medium StemPro Adipogenesis Differentiation (A1007001, Thermo Fisher Scientific, Warsaw, Poland). Cells were seeded onto 24-well plates in density of 20 × 10^4^/well. The culture media was changed every 3 days. Adipose differentiation was conducted for 14 days, and then the cells were used for further tests.

### 2.2. Proliferation Rate Assay

The proliferation rate was tested with the TOX8 Assay (TOX8 In Vitro Toxicology Assay Kit, Sigma Aldrich, Poznan, Poland). After 24 h incubation with CBD with the concentration range of 0–100 μM, the cellular medium was changed to medium containing LG, 10% FBS, 1% PS, and 10% *v*/*v* resazurin dye. The cells were then incubated for 2 h at 37 °C. The post-culture media were transferred to a 96-well plate. The fluorescence was measured at 600 nm, with 690 nm as a reference wave. Population doubling time (PDT) was calculated using an online algorithm (http://www.doubling-time.com/compute.php, accessed on 16 August 2022).

For the Clonogenic Assay, cells were seeded onto a 6-well plate at a density of 1 × 10^2^/well. The cells were treated with CBD and tunicamycin. After 7 days of incubation, cells were fixed with 4% PFA (P6148, Sigma-Aldrich/Merck, Poznan, Poland) for 30 min in the dark at RT and stained for 5 min in RT with pararosaniline (P7632, Sigma-Aldrich/Merck, Poznan, Poland). The obtained colonies (CFU) were analyzed using the formula described by Kornicka et al. (10.1111/jcmm.13914) [8].

For the scratch test, cells were seeded onto a 96-well plate at 10 × 10^4^/well. The cells were then treated with the appropriate compounds as described above, and a scar was made. Several microphotographs were taken after 0 h, 6 h, 24 h, and 48 h. The obtained data were analyzed by GraphPad Prism8 Software.

### 2.3. Visualization of Cell Organelles

A confocal microscope (Observer Z1 Confocal Spinning Disc V.2 Zeiss) was used to visualize cell organelles.

The endoplasmic reticulum was stained with 1 µM ER-Tracker Green (E34251, Invitrogen, Thermo Fisher Scientific, Warsaw, Poland). The cell medium was changed to Hankʼs Balanced Salt Solution with calcium and magnesium containing 1 µM ER-Tracker Green. Cells were incubated for 30 min at 37 °C. Then, cells were fixed with 4% PFA (Sigma-Aldrich/Merck, Poznan, Poland) as described above. Cell nuclei were stained with DAPI (Faramount Aq Mounting Medium, Dako, Santa Clara, CA, USA).

Mitochondria were stained with MitoRed dye (53271, Sigma-Aldrich/Merck, Poznań, Polska) (1:1000 in cell medium) on viable cells for 30 min. The cells were then fixed with PFA (Sigma-Aldrich/Merck, Poznan, Poland) as described above. Cells were permeabilized with 0.1% Triton X-100 (93443, Sigma-Aldrich/Merck, Poznan, Poland) solution for 15 min. The cytoskeleton was stained using atto-488-labeled phalloidin (49409, Sigma-Aldrich/Merck, Poznan, Poland) (1:800 in PBS) for 40 min in the dark at RT. The cells were washed three times in PBS. Cell nuclei were stained with DAPI (Faramount Aq Mounting Medium, Dako, Santa Clara, CA, USA).

### 2.4. Visualization of Ki-67

To visualize proliferation marker, immunostaining with Ki-67 antibody (ab15580, Abcam, Cambridge, UK) was conducted. Cells were seeded on slides in 6-well plates. After 24 h incubation with the appropriate compounds, the medium was removed, and the cells were fixed with 4% PFA as described above. Then, samples were washed with PBS and were permeabilizated with 0.05% Triton X-100 in PBS for 15 min at room temperature in the dark. Treated and untreated cells were incubated overnight with the Ki-67 antibody diluted in 10% Goat Normal Serum (#31872, Invitrogen) in PBS at 4 °C (1:1000). Cells were washed with PBS 3 times and incubated with atto-594 secondary antibody (77671, Sigma-Aldrich/Merck) diluted in PBS for 1 h in the dark in the room temperature (1:1000). The cell nucleus was stained with DAPI (Faramount Aq Mounting Medium, Dako, Santa Clara, CA, USA). Cells were observed with a confocal microscope, and the obtained data were analyzed using Image J Software.

### 2.5. Evaluation of β-Galactosidase Activation

Detection of the ageing-related lysosomal enzyme (SA-β-Gal) was performed with Senescence Cells Histochemical Staining Kit (CS0030, Sigma Aldrich, Poznan, Poland), in accordance with the instructions of the manufacturer. Cells were fixed by incubation for 6 min with 1× Fixation Buffer. The buffer was changed to staining mixture. Cells were incubated at 37 °C overnight. Cells were observed using an inverted microscope (Leica, Wetzlar, Germany).

### 2.6. Red Oil Staining

After adipogenic differentiation, the media was harvested, and the cells fixed with 4% PFA (Sigma-Aldrich/Merck) as described above. Then, cells were incubated with 60% isopropanol for 5 min at RT. Cells were stained with Oil Red O for 5 min in RT and washed twice with PBS. Cells were stained with haemotoxylin (H9627, Sigma-Aldrich/Merck) for 1 min at RT. Cells were observed using an inverted microscope (Leica, Germany).

### 2.7. Gene Expression Analysis

The total amount of RNA was isolated using EXTRAzol reagent (Blirt, Gdańsk, Poland), in accordance with the instructions of the manufacturer. The concentration and purity of the obtained RNA was measured using a nanospectrophotometer (Epoch, BioTek, Biokom, Janki, Poland). The proper amount of RNA (150 ng) was taken into a reverse transcription reaction, performed using a Takara PrimeScript RT Reagent Kit with gDNA Eraser (Perfect Real Time). Real-time PCR was performed using a SensiFast SYBR & Fluorescein Kit (Bioline, London, UK) and a CFX Connect Real-Time PCR Detection System (Bio-Rad, Hercules, CA, USA). The reaction mixture contained 7.5 µL of SensiFast SYBR & Fluorescein mix and 2.5 µL of cDNA template, respectively. The real-time PCR program was as follows: 95 °C for 2 min followed by 41 cycles at 95 °C for 15 s, annealing for 30 s (Table 1), and elongation at 72 °C for 15 s. The qpCR results were replicated in 3 independent experiments, and then the statistics were determined. Relative gene expression was normalized by the reference gene the glyceraldehyde 3-phosphate dehydrogenase (GAPDH) using the 2^−ΔΔCT^ method.

In order to conduct RT-qPCR for miRNA, a Mir-X miRNA First Strand Synthesis Kit (638315, Takara, San Jose, CA, USA) was used. Briefly, to remove a DNA template, the RNA was mixed with the DNase I, RNase-free (#EN0521), 10X reaction buffer with MgCl2 and water, and then incubated at 37 °C for 30 min. The proper volume of obtained RNA was mixed with mRQBuffer (2X) and mRQEnzyme. The reaction mixture was incubated at 37 °C for 1 h, then at 85 °C for 5 min.

The expression level of miRNA was analyzed by real-time PCR using the MicroRNA first-strand synthesis kit, in accordance with the instructions of the manufacturer. Briefly, the reaction mixture contained water, SensiFast SYBR & Fluorescein Kit (Bioline, London, UK), miRNA-specific primer (Table 2), mRQ 3′primer, and cDNA. As a reference sample, *U6F* primer and *U6R* primer were used. The relative expression level was calculated by comparison of the tested groups with the control group using the 2^−ΔΔCT^ method [19].

### 2.8. Western Blot Analysis

Protein detection was performed using the Western blot method.

Control group, tunicamycin-treated cells, and CBD-treated cells were harvested on ice with RIPA buffer (Sigma, R0278-50ML) containing 1:1000 Protease and Phosphatase Inhibitor (Sigma Aldrich, Poznan, Poland). Protein concentration was measured using the Pierce BCA Protein Assay Kit (Thermo Scientific, Waltham, MA, USA). A final concentration of 25 µg of proteins was mixed with 4 × Laemmli buffer containing β-mercaptoeatnol (Bio-Rad, Hercules, CA, USA). The samples were incubated for 5 min at 95 °C. Prepared proteins were used in the Western blot.

After SDS-PAGE electrophoresis, proteins were transfer onto polyvinylidene difluoride (PVDF) membranes (Bio-Rad, Hercules, CA, USA) and then blocked in a 5% non-fat milk solution in TBST for 1 h at room temperature. In order to prepare 10× TBST buffer, the mixture containing 0.2 M Tris base, 1.5 M NaCl, 0.1% (*w*/*v*) Tween-20, and distilled water was composed. The pH of the solution was adjusted to 7.6. In the conducted experiments, a 1× TBST buffer was used. The different membranes were incubated with primary antibodies diluted in 5% non-fat milk in 1× TBST buffer overnight (Table 3). Primary antibodies were removed, and the membranes washed with 1× TBST 5 times. Membranes were incubated for 1 h at room temperature with HRP-conjugated secondary antibodies (dilution 1:1000 in diluted 5% non-fat milk in 1× TBST buffer). The chemiluminescent signals were monitored with the ChemiDoc MP Imaging System (Bio-Rad, Hercules, CA, USA).

### 2.9. Evaluation of Oxidative Stress Factors

The concentration of reactive oxygen species in the cells was measured with a commercially available Muse Oxidative Stress kit, in accordance with the instructions of the manufacturer. The appropriate concentration of cells was suspended in 1× Assay Buffer. Cells were then incubated at 37 °C for 30 min in Muse Oxidative Stress working solution. Results were acquired with a Muse Cell Analyzer (Merck, Darmstadt, Germany).

### 2.10. Evaluation of Nictric Oxide

Detection of NO in the cells was performed with the commercially available Muse Nitric Oxide Kit. The procedure was performed in accordance with the instructions of the manufacturer. The appropriate number of cells has been suspended in 1× Assay Buffer. The Muse Nitric Oxide working solution was added to the suspension. Cells were incubated for 30 min at 37 °C, and then Muse 7-AAD working solution was added. Results were acquired with a Muse Cell Analyzer (Merck, Germany).

### 2.11. Cell Cycle Analysis

The cell cycle was tested using the Muse Cell Cycle Kit, in accordance with the instructions of the manufacturer. Treated and un-treated cells were suspended in ice cold 70% ethanol and incubated overnight in −20 °C. The cells were then centrifuged, suspended in the Muse Cell Cycle Reagent, and incubated for 30 min in the dark at RT. The cell cycle was tested using Muse Cell Analyzer (Merck, Germany).

### 2.12. Evaluation of Apoptosis

To evaluate cells apoptosis, the Muse Annexin V & Dead Cell Kit was used in accordance with the instructions of the manufacturer. Prepared cells were incubated with Muse Annexin V & Dead Cell reagent in the dark for 20 min at RT. Analysis of apoptosis were acquired with a Muse Cell Analyzer (Merck, Germany).

### 2.13. Statistical Analysis

Obtained data were analyzed by one way variance analysis (ANOVA) using GraphPad Software 8 (San Diego, USA) according to Tukey’s test. Statistically significant results (comparison of untreated cells (CTRL) to cells treated with CBD or/and tunikamycin) are marked with an asterisk, respectively, for: *p* < 0.05 (*), *p* < 0.01 (**), and *p* < 0.001 (***). Statistically significant results (comparison of treated cells with CBD (CBD1, CBD5) to cells treated with tunikamycin) are marked with a hashtag, respectively, for: *p* < 0.05 (#), *p* < 0.01 (##), and *p* < 0.001 (###). Results are presented as statistical mean SD from at least three independent experiments with three technical repetitions.

## 3. Results

### 3.1. Evaluation of Morphology, Proliferation Rate, and Adipogenic Differentiation of Affected with Tunicamycin ASCs Treated with CBD

**Figure 1:**
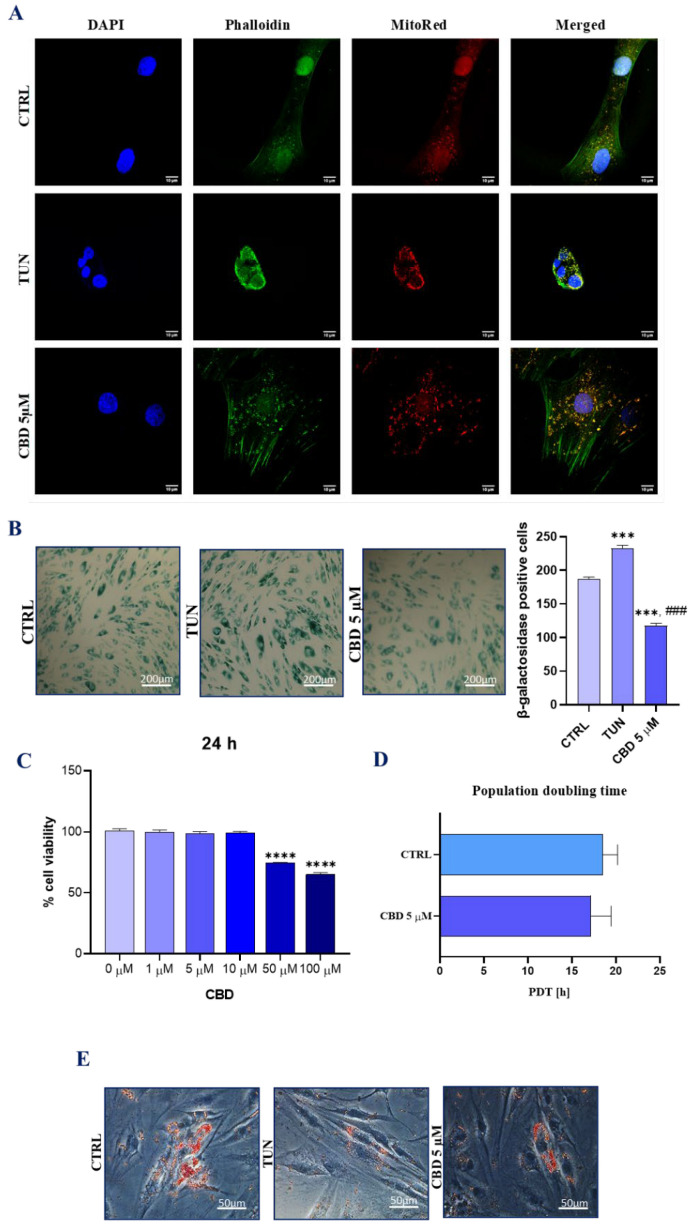

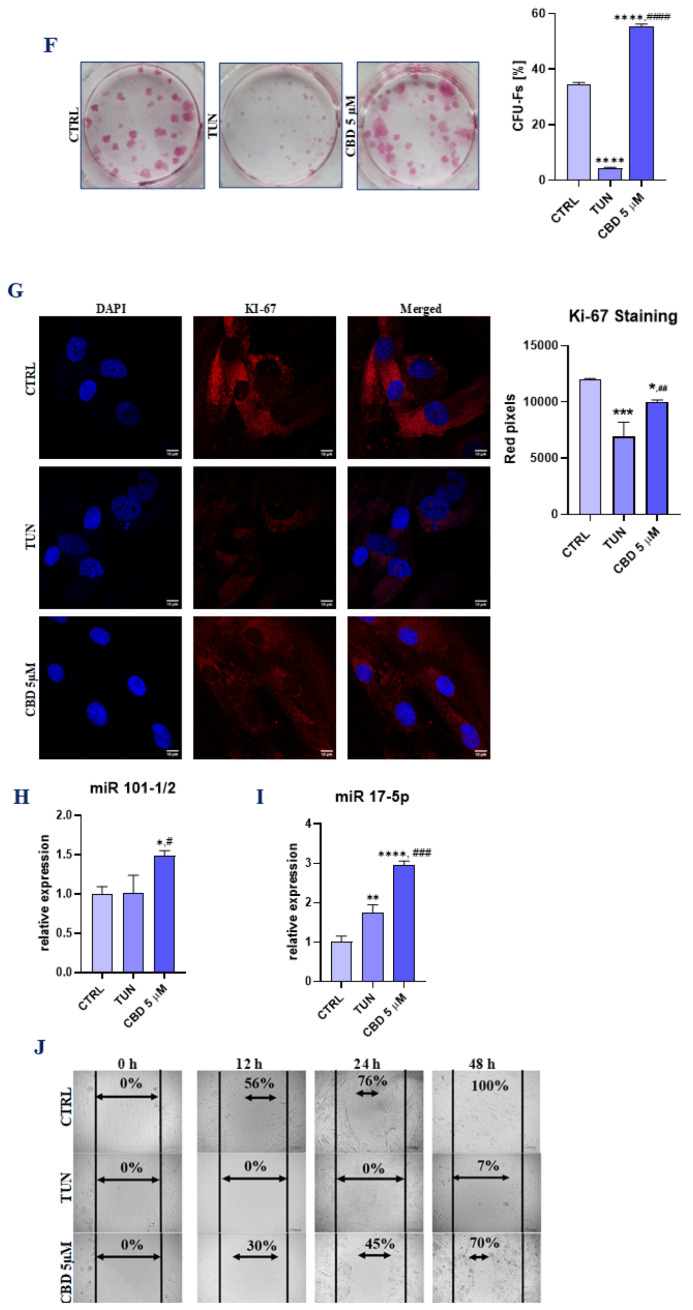
Evaluation of morphology, proliferation rate, and adipogenic differentiation of ASCs affected with tunicamycin and treated with CBD. ASCs were incubated with CBD 5 µM concentration before exposition to tunicamycin for 24 h. Morphological changes for cells pretreated with CBD 5 µM and with no treatment were estimated with confocal microscopy. ASCs were stained with DAPI, phalloidin, and MitoRed (**A**). Cellular sentence was measured using β-galactosidase. Statistical plots show statistical significance with asterisks and hashtags (**B**). Proliferation rate was measured with TOX-8 resazurin-based assay after 24 h incubation with different CBD concentrations (**C**). PDT was determined for CBD 5 µM (**D**). Correlation between lipid overload and CBD pretreatment in tunicamycin-treated ASCs was estimated with Oil Red O stain and observed with brightfield microscopy (**E**). The ability to form colonies for pretreated cells was estimated using a clonogenic test. CFU-Fa (cell colony formation) differences between groups are shown in diagram (**F**). Relative expression was measured and visualized with graphs. Immunostaining of CTRL, TUN, and CBD5µM with Ki-67 atto 594 antibody and DAPI was observed under a confocal microscope. (**G**). Mi-101-1/2 (**H**) and miR 17-5p (**I**) expression levels are shown on graphs. Cells were visualized 0 h, 6 h, 24 h, and 48 h after scarring. The black vertical lines show the edges of the scar, while the arrows show the confluence of the scar (**J**). Asterisk (*) refers to a comparison of groups treated with tunicamycin (TUN) and CBD (CBD 5 µM) to untreated healthy cells (CTRL). Hashtag (#) refers to comparison of group treated with CBD (CBD 5 µM) to group treated with tunicamycin (TUN). */# *p*  <  0.05, **/## *p*  <  0.01, ***/### *p*  <  0.001, ****/#### *p*  <  0.0001 while non-significant differences are not marked. Statistics were performed by one-way ANOVA test.

The morphology of cells was investigated using a confocal microscope (Figure 1A). Cells treated with CBD before ER stress induction with tunicamycin performed better regarding morphological features than the control group and the group treated only with tunicamycin. CBD in concentration of 5 µM improved actin density and the wide distribution of mitochondria. The appearance of the cytoskeleton and nucleus was ameliorated. Moreover, the nucleus from the CBD group had a more rounded and dense shape in comparison to that of TUN and CTRL. For cellular senescence, we used the evaluation of the well-known cellular senescent marker–β-gal. In vitro staining of ASCs showed that there were significant differences in β-gal activity between the groups. Cells treated with tunicamycin showed a higher level of β-galactosidase bonding (Figure 1B). The number of cells filled with β-gal decreased during pretreating with CBD 5µM. In order to evaluate the proliferation ratio, a TOX-8 resazurin-based assay was performed over a 24 h culture period (Figure 1C). Concentrations of 1, 5, and 10 µM had no significant effect on the proliferation level of cells. A decrease in cell viability was noticed above a 50 µM concentration of CBD. To determine the time for a cell in culture, a PDT test was performed (Figure 1D). We observed a lower PDT value for cells treated with CBD 5 µM, which suggests better proliferation properties in comparison to the control group. Additionally, we performed adipogenesis with a special-condition cell medium, and assessment of adipocyte differentiation in cell culture with Oil Red O stain (Figure 1E). The results showed better oil absorption for cells treated with CBD, before inducing ER stress with tunicamycin, than without it. Brightfield microscope images showed the spherical morphology of cells. Furthermore, we estimated the potential of CBD as a clonogenic stimulant. We observed more cell colonies on well plates that contained CBD in the cell culture medium (Figure 1F). In this study, we also determinate the expression of Ki-67, a widely used proliferation marker (Figure 1G). Ki-67 positive cells were highly detectable in the control group. Compared to the group with ER stress induction, the CBD-treated cells showed a better level of Ki-67 staining, which confirms that CBD treatment exhibits a more rapid proliferation rate. On the other hand, to measure basic cell migration parameters, we used the scratch test. ASCs treated with CBD before ER stress induction showed better migration properties (Figure 1H). Moreover, we compared the relative expression of proliferation-related miRNA in both studied groups using qRT-PCR (Figure 1I,J). Interestingly, the addition of 5 µM CBD was positively correlated with the expression of miR 101-1/2 and miR 17-5p.

### 3.2. Evaluation of Apoptosis (CBD Protects ER Stress-Induced Apoptosis in ASCs)

**Figure 2:**
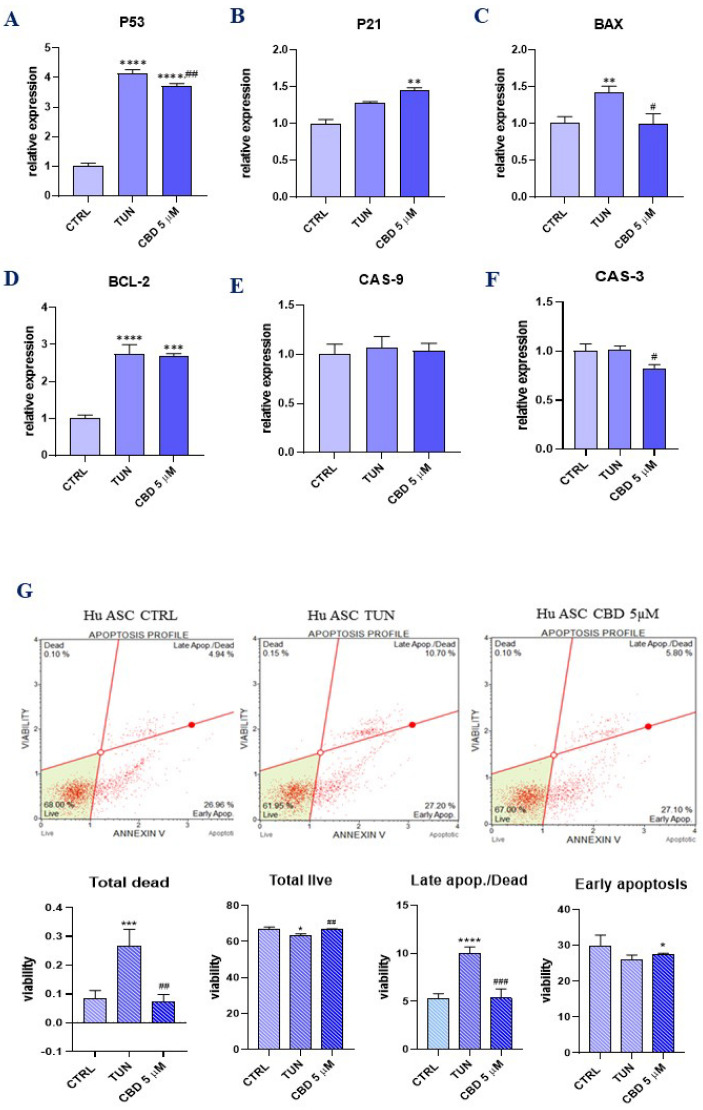
Evaluation of apoptosis (CBD protects ER stress-induced apoptosis in ASCs). Determination of apoptosis-related genes expression: *p53* (**A**), *p21* (**B**), *BAX* (**C**), *BCL-2* (**D**), *CAS-9* (**E**), and *CAS-3* (**F**). Estimation of CBD influence on apoptosis was made using Annexin-V & Dead Cell (7-AAD) flow cytometry. Apoptosis profile plots, where each is a representative figure of the three replicates of each determination. Percentage of total dead, total live, late, and early apoptosis is shown in bar charts (**G**). Asterisk (*) refers to a comparison of groups treated with tunicamycin (TUN) and CBD (CBD 5 µM) to untreated healthy cells (CTRL). Hashtag (#) refers to comparison of group treated with CBD (CBD 5 µM) to group treated with tunicamycin (TUN). */# *p*  <  0.05, **/## *p*  <  0.01, ***/### *p*  <  0.001, **** *p*  <  0.0001 while non-significant differences are not marked. Statistics were performed by one-way ANOVA test.

To determine the expression of apoptosis-related genes, we performed qRT-PCR for *p53*, *p21*, *Bcl-2*-associated X protein (*BAX*), B cell lymphoma 2 (*BCl-2*), caspase-3 (*Cas-3*), and caspase-9 (*Cas-9*). P53 protein (Figure 2A) showed reduced expression in the group treated with CBD in comparison to the TUN group with induced ER stress. Interestingly, *p21* (Figure 2B) showed overexpression for CBD pretreatment. Pro-apoptotic *BAX* was significantly decreased in the treated group (Figure 2C). In comparison, anti-apoptotic *Bcl-2* (Figure 2D) mRNA levels showed no significant changes among the groups. The same results we observed for *Cas-9* expression (Figure 1E). On the other hand, the obtained results showed that the addition of CBD decreased the expression level of *Cas-3*. (Figure 2F). What’s more, we used Muse Annexin V to obtain levels of early and late apoptosis, where the results showed a decreased number of total dead cells and cells during late apoptosis. Interestingly, for early apoptosis, there were no significant changes between the CBD 5 µM and TUN groups. The same phenomenon was observed for total live cells, where there were no differences among the groups. However, the level of total dead cells was significantly decreased in CBD pretreated cells (Figure 1G).

### 3.3. Evaluation of ER Stress (CBD Regulates PERK-, ATF6-, and IRE1-Mediated ER Stress Response)

**Figure 3:**
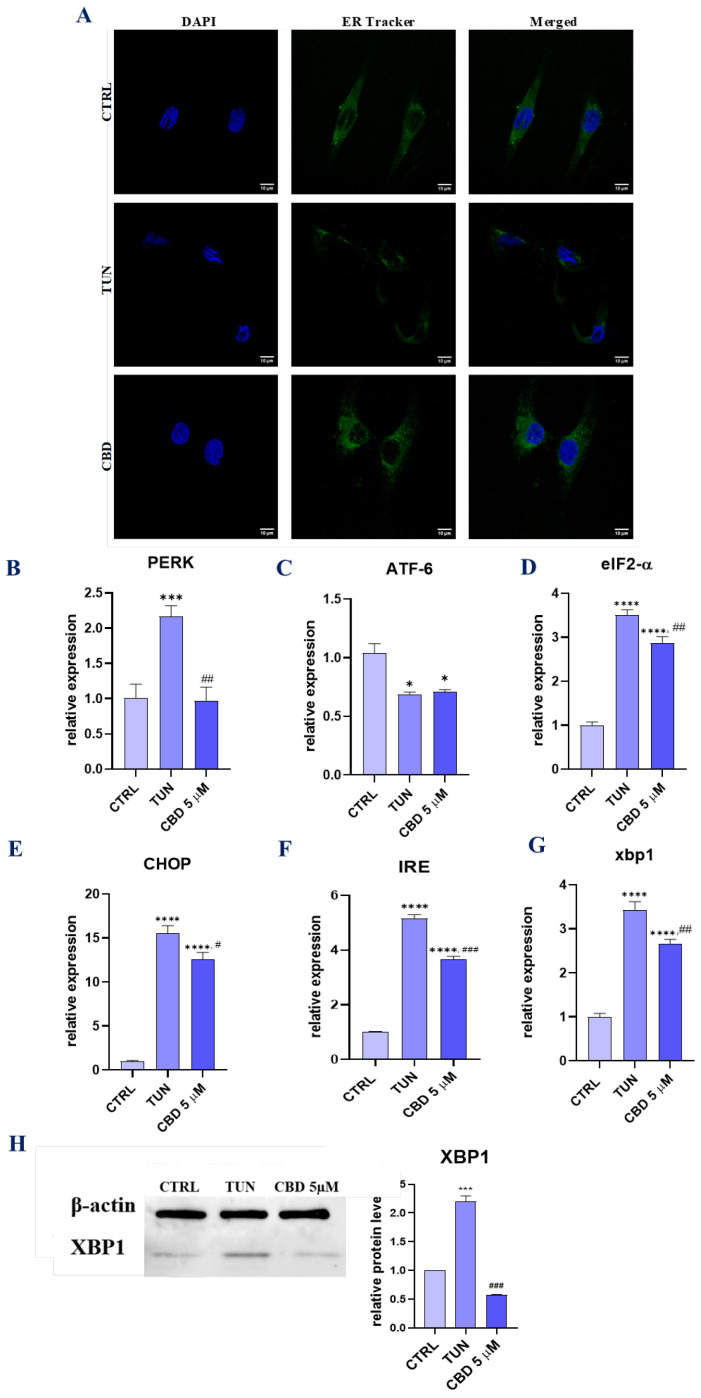
Evaluation of ER stress (CBD regulates PERK-, ATF6-, and IRE1-mediated ER stress response). To confirm the protective effects of CBD on adipose-derived stem cells we pretreated them with 5 µM CBD. ER localization in cells was visualized with ER-Tracker Green (**A**). mRNA levels of *PERK* (**B**), *ATF-6* (**C**), *eIF2-α* (**D**), *CHOP* (**E**), *IRE* (**F**), and *XBP1* (**G**) were determined using the RT-qPCR method. The levels of *XBP1* were additionally estimated using the Western blot technique (**H**). Asterisk (*) refers to a comparison of groups treated with tunicamycin (TUN) and CBD (CBD 5 µM) to untreated healthy cells (CTRL). Hashtag (#) refers to comparison of group treated with CBD (CBD 5 µM) to group treated with tunicamycin (TUN). */# *p*  <  0.05, ## *p*  <  0.01, ***/### *p*  <  0.001, **** *p* <  0.0001 while non-significant differences are not marked. Statistics were performed by using one-way ANOVA test.

To evaluate whether CBD may protect against the aggravation of tunicamycin-induced ER stress, ASCs were pretreated with CBD 5 µM for 24 h before adding ER stress inductor tunicamycin. To observe morphological changes in ER stress, we obtained a confocal microscope image with stem cells stained with DAPI and ER tracker (Figure 3A). The results showed a better fluorescent signal originating from the ER net in cells pretreated with CBD, which may suggest a better metabolic function of the endoplasmic reticulum. Additionally, we analyzed the mRNA levels of UPR-related markers, such as *PERK*, *ATF-6*, eukaryotic translation initiation factor 2 α (*eIF2-α*), *CHOP*, *IRE*, and *XBP1* (Figure 3B–G). Obtained results revealed that there were significant differences in the expressions of those genes between the groups. The overexpression of *PERK* in the TUN group was reduced in the group pretreated with CBD 5 µM. *ATF-6* relative expression was decreased for both groups, TUN and CBD 5 µM, in addition to CTRL. For all other analyzed mRNAs, including *eIF2-α*, *CHOP*, *IRE*, and *XBP1*, we observed considerably decreased expression for the CBD 5 µM group. To confirm the regulatory properties of CBD in ER stress induction we performed Western blot analysis for *XBP1* (Figure 3H), which regulates the unfolded protein response (UPR) during endoplasmic reticulum (ER) stress. In summary, the results from qRT-PCR and Western blot analysis suggest a positive role of CBD in decreasing ER stress.

### 3.4. Assessment of Oxidative Stress (CBD Attenuates Oxidative Stress in Affected with Tunicamycin ASCs)

**Figure 4:**
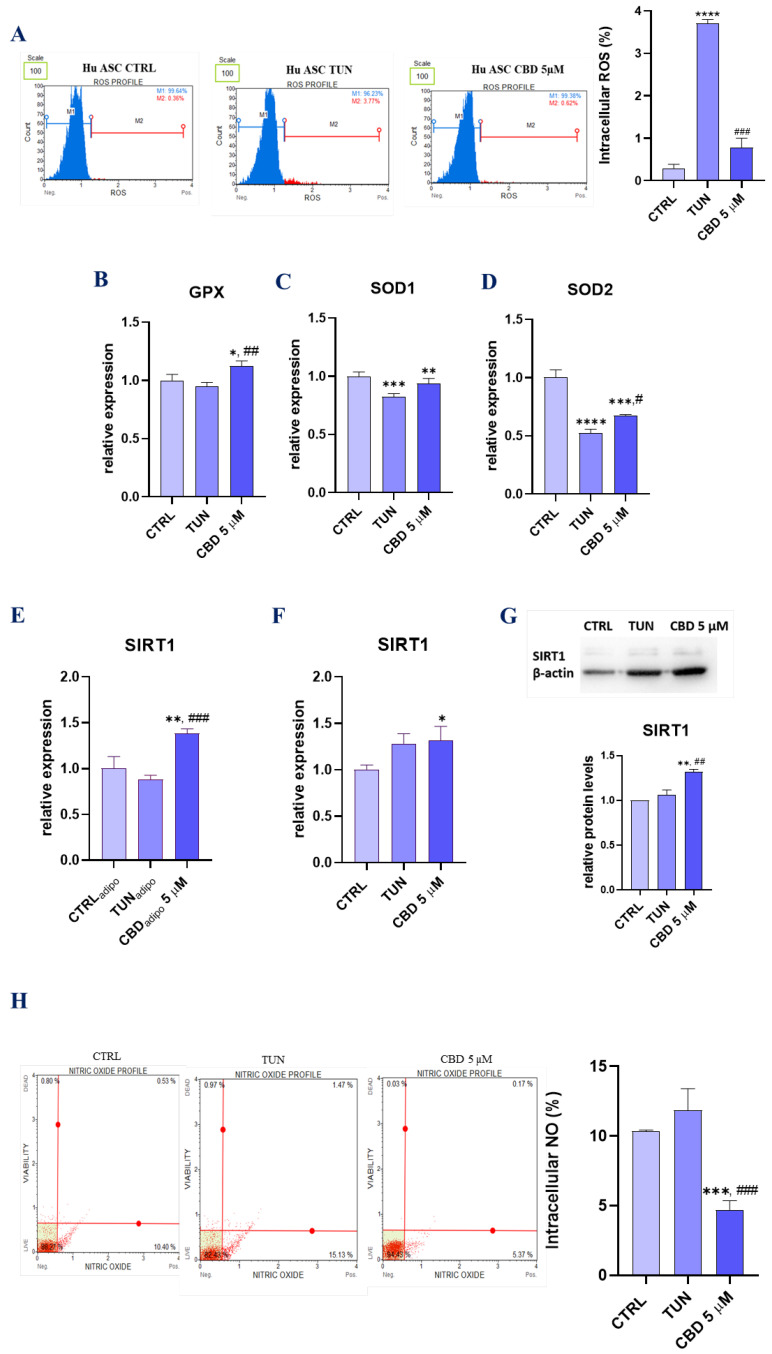
Assessment of oxidative stress (CBD attenuates oxidative stress in affected with tunicamycin ASCs). Generation of ROS induced by tunicamycin and effect of CBD on oxidative stress. The plots show the percentages of ROS − (M1) and ROS + (M2) for one representative experiment. The graph represents the summary mean percentages  ±  SD of ROS + cells from three repetitions (**A**). Effects of gene expression for *GPX* (**B**), *SOD1* (**C**), *SOD2* (**D**), *SIRT1* for native cells (**F**), and *SIRT1* for audiogenic cells (**G**) were measured by RT-qPCR. Cells were assayed with MUSE Nitric Oxide staining to measure the percentage of NO; profile plots are presented. Bar charts depicting percentage of NO (**H**). Western blot for SIRT1 estimation (**E**). Asterisk (*) refers to a comparison of groups treated with tunicamycin (TUN) and CBD (CBD 5 µM) to untreated healthy cells (CTRL). Hashtag (#) refers to comparison of group treated with CBD (CBD 5 µM) to group treated with tunicamycin (TUN). */# *p*  <  0.05, **/## *p*  <  0.01, ***/### *p*  <  0.001, **** *p* < 0.0001 while non-significant differences are not marked. Statistics were performed by one-way ANOVA test.

CBD is a well-known *Cannabis* compound with potent antioxidant properties. Hence, we investigated whether CBD in a concentration of 5 µM would decrease oxidative stress induced by tunicamycin in ASCs. We analysed ROS production with a Muse Oxidative Stress assay (Figure 4A). The results showed a decreased intercellular ROS percentage for cells pretreated with CBD. Moreover, we performed qPCR to obtain expression levels for mRNA-coding proteins involved with oxidative damage and repair (*GPX*, *SOD*, *SOD2*). Glutathione peroxidase (*GPX*), with the main role of protecting cells from oxidative damage, was effectively expressed in cells pretreated with CBD 5 µM (Figure 4B). For *SOD1* (superoxide dismutase 1), we observed no significant changes (Figure 4C). However, obtained data showed that *SOD2* (superoxide dismutase 2) mRNA expression levels were increased in the pretreated group (Figure 4D). These proteins constitute a very important antioxidant defence against oxidative stress, and their up-regulation shows good anti-oxidative stress properties of CBD. For better knowledge of CBD action in cells as an oxidative stress protector, we performed RT-qPCR for mRNA, coding sirtuin 1 (*SIRT1*) for both native and audiogenic cells. SIRT1 protein is widely known for being associated with cellular survival and involved in oxidative stress combat. Furthermore, *SIRT1* has been shown to also be involved in cell apoptosis. In our studies, the results obtained up-regulation of *SIRT1* for CBD 5 µM compared to the control group (Figure 4E). The results were confirmed with Western blot analysis (Figure 4F). Moreover, we performed the same RT-qPCR analysis for cells in audiogenic conditions. The obtained results showed even higher expression levels for CBD 5 µM in comparison to TUN (Figure 4G), which suggests that cannabidiol has protective properties for cells during their differentiation in adipocytes. Additionally, we measured intercellular nitric oxide (NO) levels by using MUSE Nitric Oxide staining (Figure 4H).

### 3.5. Assessment of the Inflammation (CBD Positively Modulates Inflammation during Stem Cell Adipogenesis)

**Figure 5:**
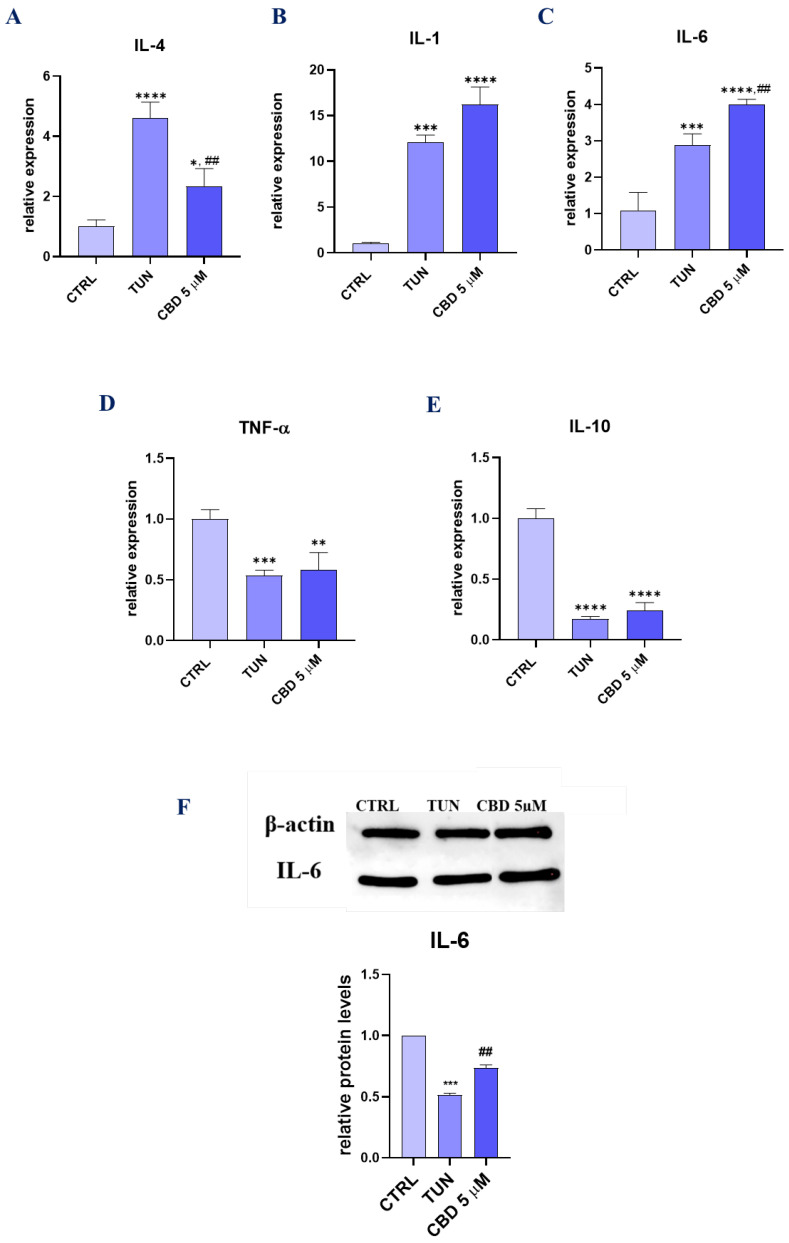

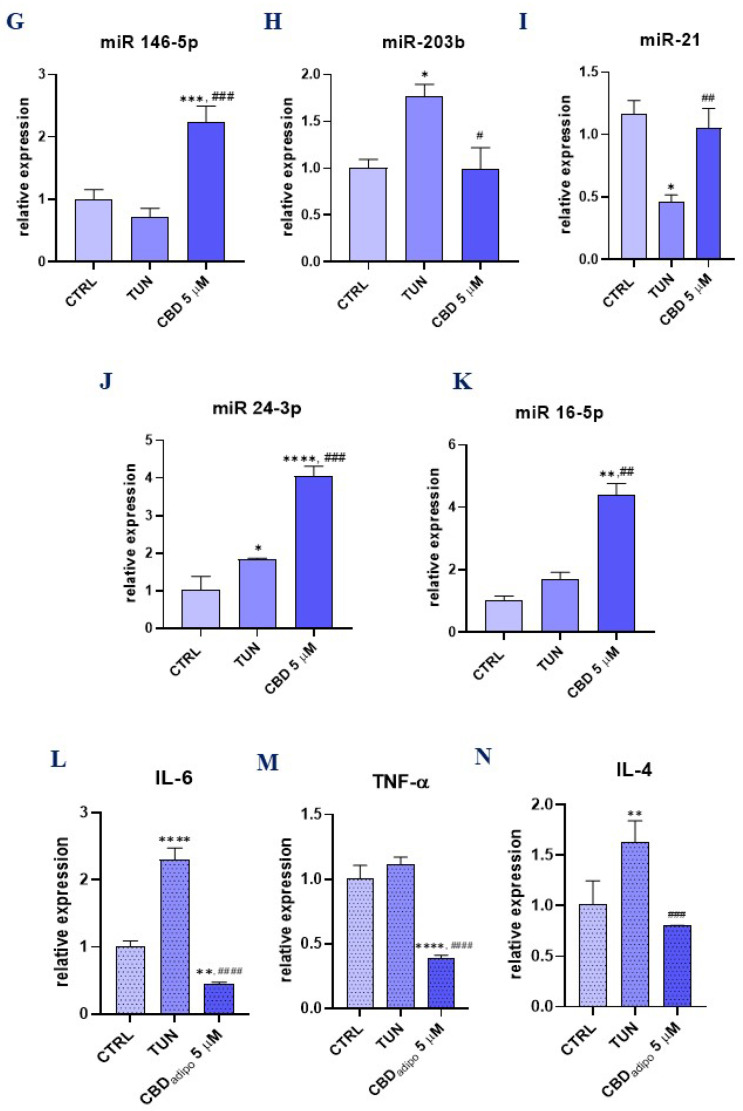
Assessment of the inflammation (CBD positively modulates inflammation during stem cell adipogenesis). Visualization of differences in expression of key inflammatory-related gens. Transcript levels of *IL-4* (**A**), *IL-1β* (**B**), *IL-6* (**C**), TNF-α (**D**), and IL-10 (**E**) were determined with RT-cPCR. IL-6 protein concentration was estimated using Western blot technique (**F**). Micro-RNA expression levels were established for *miR 146-5p* (**G**), *miR-203b* (**H**), *miR-21* (**I**), *miR 24-3p* (**J**), and *miR 16-5p* (**K**). The expression of three inflammatory-related genes, *IL-6* (**L**), *TNF-α* (**M**), and *IL-4* (**N**), for adipogenic cells was determined with RT-qPCR. Asterisk (*) refers to a comparison of groups treated with tunicamycin (TUN) and CBD (CBD 5 µM) to untreated healthy cells (CTRL). Hashtag (#) refers to comparison of group treated with CBD (CBD 5 µM) to group treated with tunicamycin (TUN). */# *p*  <  0.05, **/## *p*  <  0.01, ***/### *p*  <  0.001, ****/#### *p*  <  0.0001 while non-significant differences are not marked. Statistics were performed by one-way ANOVA test.

Mechanisms underlying inflammation and ER stress are widely known. In this paper, we investigated if CBD would reduce inflammation that was a response to ER stress induced by tunicamycin. In order to elucidate the alternations in the expression of inflammation-related cytokines and miRNA, RT-qPCR was performed for both progenitor and differentiated adipocyte cells. The analysis showed down-regulation of mRNA encoding interleukins such as *IL-4*, *IL-10*, and *TNF-α* (tumour necrosis factor). Interestingly, we observed up-regulation of mRNA coding *IL-1β* and *IL-6* in a group pretreated with CBD. (Figure 5A–E). For IL-6, Western blot analysis was performed. Interestingly, results obtained decreased levels of that cytokine in the CBD 5 µM group (Figure 5F). For a better understanding of CBD action as an inflammatory regulator, we performed the analysis of the microRNAs correlated with inflammation. *MiR 146-5p* was significantly more overexpressed in pretreated cells than in CTRL and TUN (Figure 5G). For *miR-203*, we observed opposite results, where CBD decreased the level of that microRNA’s expression (Figure 5H). Both anti-inflammatory *miR-21* (Figure 5I) and *miR 24-3p* (Figure 5J) were up-regulated for pretreated groups. What is more, in pretreated cells, we observed up-regulation of the main miRNA associated with inflammation. *miR 16-5p*, which is reported to down-regulate the pro-inflammatory factors, such as interleukin IL-6 and tumour necrosis factor-α (TNF-α), was significantly up-regulated in ASCs CBD 5 µM (Figure 5K). Additionally, we performed RT-qPCR for cells during adipogenesis (Figure 5L–N). Using CBD 5 µM as pretreatment, we observed the down-regulation of *IL-6* (Figure 5L) and *TNF-α* (Figure 5M). Interestingly, the results obtained for *IL-4* (Figure 5N) showed significant down-regulation of that gene. These results may suggest that CBD is a good inflammatory regulator for different cells and can obtain healthy adipocytes free from inflammation by targeting different inflammatory factors.

## 4. Discussion

The ECS system participates in the control of food intake, appetite, and energy balance. Endocannabinoids and their receptors are present in AT and modulate its metabolism via interaction with hormones, adipokines, insulin, and transcription factors. However, many of these pathways are impaired during metabolic disorders, which contributes to ECS deregulation and disease progression. ECS action is mediated through CB1 receptors, which are present on human and rodent adipocytes and its knock down leads to leanness, resistance to diet-induced obesity, and enhanced leptin-sensitivity in mice [20]. On the other hand, expansion of AT requires proliferation and differentiation of ASCs, and, for that reason, these cells may represent potential therapeutic target against metabolic disorders. Although, many studies, including our own, indicated that obesity, diabetes, and metabolic syndrome significantly impair the cytophysiological properties of ASCs, which questions their clinical administration in autologous settings. If ASCs’ regenerative properties can be improved, it could not only potentially save time and costs but also increase therapy effectiveness. The decreased regenerative properties of these cells are associated with excessive accumulation of oxidative stress factors and ER stress due to intrinsic stimuli coming from the unfriendly, inflamed microenvironment of AT. Thus, solutions targeting ASCs that are able to restore their functionality both in vitro and in vivo are now under investigation.

One of them focuses on the modulation of ECS, as it was proven to modulate multiple cell functions including proliferation, differentiation, apoptosis, and stemness. For that reason, in the presented study, we have investigated the protective effects of CBD, a non-euphoric compound of *Cannabis sativa*, against ER stress-induced impairment of ASCs. We aimed to verify the hypothesis, if preconditioning with CBD before transplantation could improve cytophysiological properties of ASCs and, as a consequence, enhance their therapeutic efficacy.

We have found the dosage of 5 µM to be most potent, which stands in agreement with Fellous et al. [21], who revealed that CBD and other cannabinoids promote viability and functional adipogenesis of bone-marrow-derived mesenchymal stem cells at the concentration of 5 µM. Similar findings were noted by Schmuhl et al. [22], who found that CBD at 3 μM or even 10 nM improves the migration of ASCs. We have found that cells treated with CBD maintain their proper morphology, and the actin cytoskeleton and mitochondrial net were comparable to the control, untreated cells. Furthermore, CBD significantly reduced the number of senescent cells. CBD produced a robust effect on CFU-Fs and maintained the expression of Ki-67 antigens. Our results are consistent with the findings of Luo et al. [23], who discovered that CBD enhanced the proliferation of human brain endothelial cells. On the other hand, the anti-proliferative and pro-apoptotic properties of CBD were described for multiple cancer cell lines [24,25], which indicates that it may have potential as a new therapeutic target in cancer. In the presented study, we have found that CBD pretreatment reduced the expression of pro-apoptotic genes—*p53*, *p21*, *BAX*, and *Casp-3* at a concentration of 5 µM. Reduction in apoptosis by CBD was also proven by cell cycle analysis, which revealed that CBD reduced the total number of dead cells. The anti-apoptotic effects of CBD were also described by Libro et al. [26], although in human-gingival-derived MSC. The observed phenomenon probably results from CB1R activation, as it was shown to be crucial for MSC survival and differentiation [27,28]. A couple of hours after ASCs’ transplantation, an unfavourable microenvironment can trigger their apoptosis, so development of new strategies to improve ASCs’ stemness and survival are necessary. Our study confirmed that pretreatment of ASCs with CBD increased their growth kinetics and prevented ER stress-induced apoptosis.

Metabolic disorders are related to oxidative damage, collapse of antioxidant defence with a significant down-regulation of antioxidants such as superoxide dismutase or glutathione peroxidase. Reactive oxygen species (ROS) and reactive nitrogen species (RNS), including nitric oxide (NO), accumulate in the microenvironment or directly within the cell body, inducing irreversible damage to macromolecules including lipids, proteins, and DNA peroxidation. Multiple studies indicated the inference of oxidative stress in diabetes pathogenesis and its complications including stroke, neuropathy, retinopathy, and nephropathy [29]. Following treatment of ASCs with CBD, the levels of produced ROS were significantly reduced compared to untreated cells. Elevated ROS levels in diabetes partially result from decreased production of potent antioxidants, including CAT, SOD, and GPX. Here, we have found that CBD is able to enhance the expression of SOD and GPX and, therefore, enhance cellular survival. Notably, antioxidants have already been shown prospective in the treatment of diabetes and metabolic syndrome.

Here, we have found that CBD effectively protects against ER stress development in vitro. It reduced the expression of *PERK*, *ATF-6*, *eIF2-α*, *CHOP*, *IRE-1*, and *XBP1*, key mediators in ER stress pathway. Under metabolic syndrome or T2D condition, cells abundantly accumulate unfolded or misfolded proteins in the ER lumen, which directly leads to the activation of these marker genes [30]. The expression of three key UPR signal activators, *IRE-1*, *ATF-6*, and *XBP1*, was significantly down-regulated in cells pretreated with CBD. The main goal of UPR is to restore ER protein homeostasis and ensure cell survival. However, its prolonged activation, caused by unmitigated severe ER stress, triggers a signaling switch resulting in cell death and apoptosis [31] via CHOP activation. Under non-stress conditions, the expression of CHOP is reduced, but during ER stress it becomes activated and imitate apoptosis. ER stress is well-known for its role in the pathogenesis of diabetes, as its leads to insulin resistance and pancreatic beta-cell loss [32]. It was demonstrated that high-fat feeding and obesity induce ER stress in the liver, which suppresses insulin signaling via c-Jun N-terminal kinase activation [33]. For that reason, the obtained results may contribute to a better understanding of ER stress regulation and may support the design of novel therapies based on CBD application, to prevent the development of ER stress during metabolic disorders.

Inflammatory responses play a central role in the ethiopathogenesis of many metabolic disorders, and stem-cell-based therapies may provide a novel approach to enhance the regenerative process. Multiple studies have shown that ASCs exert immunomodulatory actions, e.g., regulate the immune cells fate, reduce apoptosis, and secrete a wide range of cytokines. We analysed the expression profile of both pro- and anti-inflammatory cytokines. Most significant results were obtained for *IL-4* expression in both CBD groups. It was shown that IL-4 inhibits adipogenesis by down-regulating the expression of peroxisome proliferator-activated receptor-γ and CCAAT/enhancer-binding protein-α as well as promoting lipolysis by enhancing the activity and translocation of hormone sensitive lipase (HSL) in mature adipocytes [34]. On the other hand, cytokine exerts anti-inflammatory effects by reducing the production and activities of IL-1β, TNF-α, and IL-6 [35,36]. What is more, animal studies revealed that IL-4 promotes insulin sensitivity and glucose tolerance and inhibits lipid deposits [37]. Pretreatment of cells with CBD increased the expression of *IL-1β*, *IL-6*, and *IL-10*. The obtained data clearly indicate that CBD is capable of modulating the secretory activity of ASCs treated with ER stress inducer. In the next step of the experiment, to further explore the effects of CBD on cytokine secretion, CBD was added to mature adipocytes differentiated from ASCs. In that scenario, the observed results were more pronounced. We have found that CBD pretreatment significantly diminished the expression of *IL-6*, *IL-4* and *TNF-α* in ASCs following ER stress induction. The present findings provide further support for the anti-inflammatory effects of CBD published previously [38,39,40,41]. Additionally, the expression of miRNA involved with inflammatory response was investigated. We have found that CBD at concentration of 5µM significantly reduced the expression of *miR-203b*, which correlates with the anti-inflammatory properties of CBD. As shown by Zhang et al. [42], down-regulated *miR-203* attenuated *IL-1β*, *IL-6*, and *TNF-α* activation in TRAF6-treated human renal mesangial and tubular epithelial cells. Furthermore, Cai et al. [43] revealed that *miR-203* suppression inhibits lipopolysaccharide-induced human intervertebral disc inflammation and degeneration by up-regulating estrogen receptor α. Similarly, CBD treatment enhanced expression of miR-24-3p, which inhibits inflammatory responses and attenuates IL-1β-induced cell injury [44,45,46]. In line with this, CBD also enhanced expression of *miR146-5p*, which is known for anti-inflammatory action and controls cytokine production by macrophages [47,48]. We have also found an increase in the expression of *miR-21* and *miR-16-5p*, which suppress inflammation via modulation of macrophages’ activity [49,50,51]. Overall, our data indicate that CBD treatment enhances the anti-inflammatory properties of cytophysiologically impaired ASCs.

## 5. Conclusions

The regenerative potential of ASCs in the treatment of multiple disorders lies in their differentiation, migration, and secretory activity. However, these conditions impair the cytophysiological properties of ASCs, limiting their application in autologous therapies. What is more, impaired ASCs in vivo suffer from reduced multipotency and produce a vast number of inflammatory cytokines and oxidative stress factors, which in turn contributes to disease progression. Hence, development of strategies that reverse their senescence and ageing, and, as a consequence, restore regenerative properties are strongly desirable. To our knowledge, this is the first report on the impact of CBD pretreatment on metabolically impaired ASCs suffering from ER stress. Our current study revealed that CBD modulates ASCs metabolism by promoting their growth kinetics, multipotency, and viability, which due to enhanced ER stress were strongly limited. Taking into account that CBD lacks psychopharmacological activity, further studies aiming at unravelling its influence on different stem cells populations are recommended and justified. Further studies on the effects of CBD on ASCs could explore other measures of its regenerative capacity than studied in the presented research. Furthermore, unravelling the precise molecular mechanisms of action via CBD that protect ASCs against cytophysiological impairment would be valuable. While our findings are supported by the existing literature, our research was not free of limitations. An important drawback is that we did not explore which cannabinoid receptors are responsible for the observed effects of CBD. Thus, further experiments utilizing agonists and antagonists of cannabinoid receptors are necessary to elucidate which of them are involved in CBD’s way of action. Taking into consideration that ASCs are nowadays a commonly applied tool in regenerative medicine, the ability to enhance their stemness and regenerative potential may contribute not only to more effective therapies but also to significantly reducing the costs associated with their isolation and expansion.

## Figures and Tables

**Table 1:** Sequences of primers used in qPCR with annealing temperatures.

Gene	Primer Sequence (5′->3′)	Annieling Temperature [°C]
*BAX*	F: ACCAAGAAGCTGAGCGAGTGTC	60.4
	R: ACAAAGATGGTCACGGTCTGC	
*BCL2*	F: ATCGCCCTGTGGATGACTGAG	60.4
	R: CAGCCAGGAGAAATCAAACAGAGG	
*p21*	F: TGCCGAAGTCAGTTCCTTGT	60.4
	R: GTTCTGACATGGCGCCTCC	
*p53*	F: AGTCACAGCACATGACGGAGG	60.4
	R: GGAGTCTTCCAGTGTGATGATGG	
*Cas3*	F: GCGGTTGTAGAAGTTAATAAAGGT	62.8
	R: CGACATCTGTACCAGACCGAG	
*Cas9*	F: TTGGTGATGTCGAGCAGAAAG	61.4
	R: CCAGGGTCTCAACGTACCAG	
*GPX*	F: CTCCGGAACAACAGCCTTCT	51
	R: GGAAAGGGGTCTGTGATGGG	
*SOD1*	F: GACCATTGCATCATTGGCCG	51
	R: CAAGCCAAACGACTTCCAGC	
*SOD2*	F: GGAGCGGCACTCGTGG	52
	R: CAGATACCCCAAAGCCGGAG	
*SIRT1*	F: ACAGGTTGCGGGAATCCAAA	66
	R: GTTCATCAGCTGGGCACCTA	
*IL-1β*	F: AAACAGATGAAGTGCTCCTTCCAGG	66
	R: TGGAGAACACCACTTGTTGCTCCA	
*IL-6*	F: TCCTTCTCCACAAACATGTAACAA	66
	R: ATTTGTGGTTGGGTCAGGGG	
*TNF α*	F: AGTGACAAGCCTGTAGCCCA	62.4
	R: GTCTGGTAGGAGACGGCGAT	
*IL-4*	F: CTTTGCTGCCTCCAAGAACAC	62.4
	R: GCGAGTGTCCTTCTCATGGT	
*IL-10*	F: AGACAGACTTGCAAAAGAAGGC	65
	R: TCGAAGCATGTTAGGCAGGTT	
*PERK*	F: TGCTCCCACCTCAGCGAC	67
	R: TTTCAGGATCCAAGGCAGCA	
*eIF2-α*	F: ATGTTTCAGCCAAGCCCAGA	61.4
	R: ACCAGGGGATCTACCACCAA	
*CHOP*	F: TAAAGATGAGCGGGTGGCAG	64.5
	R: GGATAATGGGGAGTGGCTGG	
*ATF6*	F: ACCTCCTTGTCAGCCCCTAA	65.9
	R: CACTCCCTGAGTTCCTGCTG	
*IRE1*	F: CGGCCTCGGGATTTTTGGA	66.6
	R: AGAAAGGCAGGCTCTTCCAC	
*XBP1*	F: CGCGGATCCGAATGAAGTGAGGCCAGTG	62.8
	R: GGGGCTTGG TATATATGTGG	

*BAX: BCL-2*-associated X protein; *BCL-2*: B-cell lymphoma 2; *p21*: cyclin-dependent kinase inhibitor 1A; *p53*: tumour suppressor *p53*; *Cas3*: Caspase-3; *Cas9*: Caspase-9; *GPX*: glutathione peroxidase; *SOD1*: superoxide dismutase [Cu-Zn]; *SOD2*: superoxide dismutase 2; *SIRT1*: sirtuin 1; *IL-1β*: interleukin 1β; *IL-6*: interleukin-6; *TNF α*: tumour necrosis factor α; *IL-4*: interleukin-4; *IL-10*: interleukin-10; *PERK*: protein kinase RNA-like ER kinase; *eIF2-α*: eukaryotic initiation factor 2 α; *CHOP*: C/EBP homologous protein; *ATF6*: activating transcription factor 6; *IRE1*: inositol-requiring enzyme 1; *XBP1*: X-box binding protein 1.

**Table 2:** Sequences of microRNA primers used in qPCR.

Primer miRNAs	Primer Sequence (5′->3′)
miR101-1/2	TACAGTACTGTGATAACTGAA
miR17-5p	CAAAGTGCTTACAGTGCAGGTAG
miR16-5p	TAGCAGCACGTAAATATTGGCG
miR-203b	TTGAACTGTTAAGAACCACTGGA
miR-21	TAGCTTATCAGACTGATGTTGA
*miR 24-3p*	TGGCTCAGTTCAGCAGGAACAG
miR 146-5p	TGAGAACTGAATTCCATGGGTT

**Table 3:** List of antibodies used in study.

Antibodies	Concentrations	CAT Numbers	Company
β actin	1:1000	orb10033	Biorbyt
SIRT1	1:1000	ARP32386	Aviva
IL-6	1:1000	ab6672	Abcam
XBP1	1:1000	ARP31440_P050	Aviva

SIRT1: sirtuin 1; IL-6: interleukin-6; XBP1: X-box binding protein 1.

## Data Availability

The datasets generated during and/or analysed during the current study are available from the corresponding author on reasonable request.
